# Association Between Personal, Behavioral, Psychological, Biochemical and Molecular Biomarkers with Illness Count in a Sample of Mexican Individuals

**DOI:** 10.3390/ijms27052408

**Published:** 2026-03-05

**Authors:** Aniel Jessica Leticia Brambila-Tapia, Juan Manuel Ponce-Guarneros, Ana Míriam Saldaña-Cruz, Saúl Ramírez-De los Santos, Heriberto Jacobo-Cuevas

**Affiliations:** 1 Departamento de Psicología Básica, Centro Universitario de Ciencias de la Salud (CUCS), Universidad de Guadalajara, Guadalajara 44340, Jalisco, Mexico; saul.rdelossantos@academicos.udg.mx; 2 Group of Assessment of Prognosis Biomarkers in Autoimmune Disorders, Centro Universitario de Ciencias de la Salud (CUCS), Universidad de Guadalajara, Guadalajara 44340, Jalisco, Mexico; juan.ponce4091@academicos.udg.mx (J.M.P.-G.); ana.saldanac@academicos.udg.mx (A.M.S.-C.); 3 Instituto de Terapéutica Experimental y Clínica (INTEC), Departamento de Fisiología, Centro Universitario de Ciencias de la Salud (CUCS), Universidad de Guadalajara, Guadalajara 44340, Jalisco, Mexico; 4 Instituto de Investigación en Ciencias Biomédicas (IICB), Centro Universitario de Ciencias de la Salud (CUCS), Universidad de Guadalajara, Guadalajara 44340, Jalisco, Mexico

**Keywords:** illness count, Mexican individuals, biomarkers, inflammation, oxidative stress, psychological variables, biochemical variables

## Abstract

The sum of diseases has been associated with many personal, behavioral, and psychological variables as well as with many biochemical, inflammatory, oxidative stress, and epigenetic biomarkers. However, the search for the association between some of these biomarkers and illness count is limited, particularly in Mexican individuals. (1) To determine the associations between personal, behavioral, biochemical, and molecular factors with the illness count in Mexican individuals, globally and segmented by sex, and (2) to determine the intercorrelation among the studied biomarkers. Mexican adults were invited to participate, and many personal, psychological, and biochemical variables were measured; in addition, the systolic blood pressure, body mass index, and waist-to-hip ratio were obtained. The self-report of 28 health conditions was measured, and the detection of 3 (diabetes, hypertension, and dyslipidemia) conditions was obtained with biochemical analyses and blood pressure measurement; with these reports, we obtained the variable illness count. A total of 157 individuals were included, of whom 83 (52.9%) were women; the median age and range were 24 (18–58) years old, and all participants were Mexican mestizo individuals. Women showed a higher number of self-reported/detected diseases than men. The multivariate analyses revealed that female sex, age, having children, risky eating behavior, poor sleep quality, systolic blood pressure, and lower levels of IL-10 were significantly correlated with the illness count. In the women’s sample, lower levels of IL-10, less free time, monthly earnings, and depression were positively correlated with illness count. In the men’s sample, the age, systolic blood pressure, poor sleep quality, 8-OHdG, IL-6, and plateletcrit, in addition to positive relations with others, were variables positively correlated with illness count. In the correlations of the studied biomarkers, we found that IL-10, TNF-α, and IL-8 showed a high positive correlation among them; in addition, inflammatory and oxidative stress biomarkers showed low significant correlations among them. Many personal, biochemical, and psychological factors are associated with the number of diseases, while the associated biomarkers differed in each sex, highlighting the role of IL-10, 8-OHdG, IL-6, and plateletcrit.

## 1. Introduction

Previous reports have shown that the illness count that a person experiences is linked with a wide variety of factors, including personal, behavioral, and psychological ones. Among the personal and behavioral variables associated with the illness count are the female sex, age, schooling, socioeconomic position, and lower sleep quality. Psychological factors associated with the illness count are anxiety, depression, and stress symptoms, psychological well-being, emotional intelligence, and maladaptive stress coping strategies [1,2].

In addition, several molecular biomarkers have been linked to the presence of chronic diseases, including inflammatory and epigenetic biomarkers. Previous studies have shown that epigenetic biomarkers related to age acceleration are related with conditions such as cardiovascular disease-related mortality in the diabetes population, and the risk to present cancer and diabetes [3,4]. Another report demonstrated an association between genetic and epigenetic modifications with protein biomarkers commonly linked with chronic diseases. Additionally, this study suggested that biomarker expression may also influence epigenetic patterns, leading to a bidirectional relationship between genetic and epigenetic modifications and biomarker expression, particularly for inflammatory biomarkers [5]. In this sense, other reports have shown that inflammatory biomarkers such as the neutrophil-to-lymphocyte ratio, along with other complete blood cell count (CBC)-derived inflammatory biomarkers, have been associated with mortality, incident cardiovascular disease, and chronic conditions, including asthma and respiratory disease [6,7,8]. Additionally, other biochemical variables, such as the platelet count, have been associated with all-cause mortality in both sexes in a large cohort of the Danish population [9].

However, despite all these findings, to date, no studies have investigated the combined relationship between personal and psychological variables along with inflammatory, oxidative stress, epigenetic, and biochemical biomarkers in relation to disease burden in a non-random sample of Mexican adults. These analyses could increase the knowledge about disease presence and its correlated variables adjusted by the most associated confounders.

In a previous report performed by the research team [10], we searched for the associations between the same studied variables included in this report, but with global DNA methylation; the difference between the reports is that in the present one, we searched for the association between these variables and illness count, which consisted of self-reported and detected diseases. Both objectives were a part of the same project.

Therefore, the objectives of the present study were (1) to determine the associations between personal, behavioral, psychological, biochemical, and molecular factors with the illness count in a non-random sample of Mexican adults, and (2) to assess the interrelationships among the biomarkers evaluated.

## 2. Results

A total of 157 individuals consented to participate and were included in the analyses. Detailed descriptive statistics and sex-based comparisons are presented in Table 1. All the included participants were Mexican mestizo individuals. Women reported a slightly higher (with a borderline *p*-value) number of illnesses and perceived stress compared with men, and the most frequent diseases reported were headache, gastritis, colitis, skin problems, and gastrointestinal infections, while the most frequently detected disease was dyslipidemia, being higher in men than in women (Supplementary File).

In contrast, men showed higher frequencies of alcohol and illicit substance use, a higher frequency of out-of-home food consumption, as well as higher levels of positive emotions, comprehensibility, and manageability. With respect to inflammatory and oxidative stress biomarkers, no significant sex differences were detected except for interleukin-6 (IL-6), which was higher in women than in men. Differences in biochemical variables were also observed, with higher levels of glucose, hemoglobin, and global DNA methylation levels in men than in women, and higher levels of platelets and plateletcrit in women than in men. In addition, men showed higher values of WHR and both blood pressures.

With respect to the biomarkers’ distribution, the percentage of outliers ranged from 0 to 19.9%. Being higher for IL-10 (19.9%), followed by IL-8 (14.6%), these percentages coincide with the large intersubject variation observed in these cytokines’ detection [11].

### 2.1. Bivariate Correlations with the Illness Count

Regarding the total sample and the sex-stratified analyses, Table 2 summarizes the statistically significant bivariate correlations between the studied variables and illness count. In the overall sample, we observed that male sex and free time showed a very weak negative correlation with illness count; while having children, out-of-home food consumption, excessive food consumption, and BMI showed a very weak positive correlation with this variable.

In the women’s sample, in addition to some of the previous correlations detected in the total sample, positive significant and weak correlations were found with depression and TE, while a significant negative weak correlation with TNF-α was also detected. In the men’s sample, we also observed a significant negative weak correlation with dietary quality and weak positive correlations with age, schooling, WHR, erythrocytes, hematocrit, and 8-OHdG.

### 2.2. Multivariate Regression for Illness Count

In the overall multivariate analysis for the global sample (Table 3), several variables showed significant correlations with illness count. Among the sociodemographic variables, male sex showed a significant negative association with it, and having children showed a positive association. Likewise, the variables out-of-home food consumption and SBP also demonstrated significant positive associations with illness count. Finally, the psychological variable self-motivation showed a borderline negative correlation with the dependent variable.

Among women, the first variable included in the model was IL-10, which was negatively associated with illness count and contributed to the greatest change in R^2^. Among the sociodemographic variables, free time was negatively associated with illness count, whereas monthly income was positively associated with it. Regarding psychological variables, depression showed a positive correlation with the dependent variable (Table 3).

Finally, in the men subsample, age, SBP, and the psychological variable “positive relations with others” showed positive correlations with illness count, while sleep quality showed a borderline negative correlation with it. Additionally, the biochemical variable plateletcrit was positively associated with the dependent variable, while the biomarkers IL-6 and 8-OHdG were positively correlated with it (Table 3).

The multivariable regression models showed satisfactory fit. In the total sample, R = 0.462 and R^2^ = 0.21, indicating that predictors accounted for 21.0% of the variance in illness count. Among women, the model yielded R = 0.538 with R^2^ = 0.29 (29.0% of the variance explained), whereas among men, R = 0.671 with R^2^ = 0.45 (45.0% explained).

### 2.3. Bivariate Correlations Among the Biomarkers

**Figure 1:**
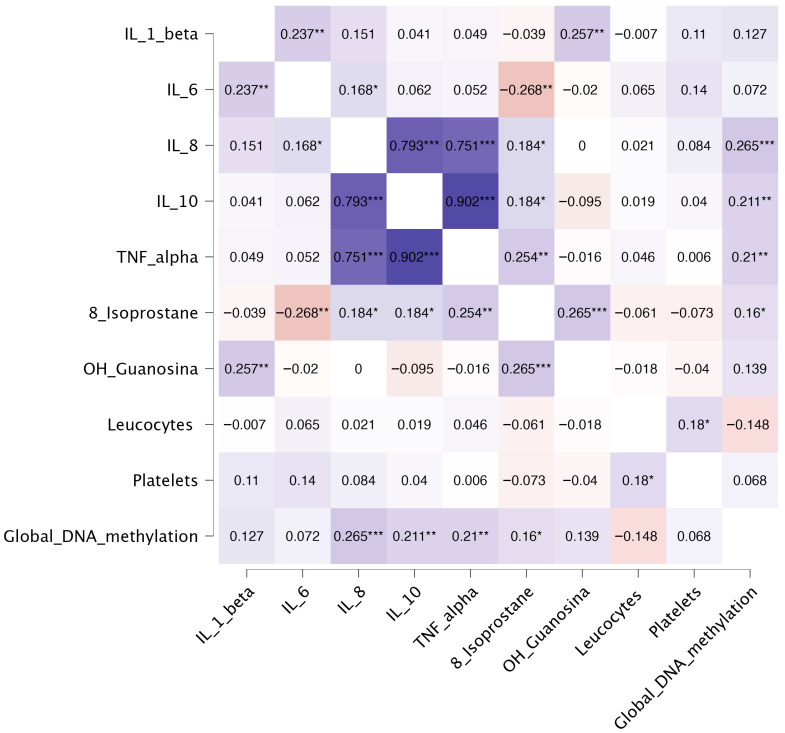
Heatmap showing the correlation coefficients between the studied biomarkers. The heatmap shows Spearman’s rank correlation coefficients between studied biomarkers. Values range from −1 (red, negative association) to +1 (blue, positive association), with color intensity reflecting correlation magnitude. The heatmap was generated using JASP (version 0.95.1). *, *p* < 0.05, **, *p* < 0.01, ***, *p* < 0.001.

In this study, we analyzed the significant bivariate correlations among biomarkers in the total sample (Table 4 and Figure 1) and in the sex-segmented subsamples (Table 5). Focusing first on the total sample, the correlation analysis revealed many correlations across inflammatory, oxidative stress, biochemical, and epigenetic biomarkers. Within the inflammatory panel, IL-8, IL-10, and TNF-α formed a tightly interconnected cluster, showing strong positive correlations with one another (r = 0.751–0.902). IL-6 showed weak positive correlations with IL-8 and IL-1β, and a weak negative correlation with 8-isoprostane, while IL-1β additionally correlated weakly with 8-OHdG. Oxidative stress markers demonstrated correlations with inflammatory biomarkers: 8-isoprostane correlated weakly and positively with IL-8, IL-10, and TNF-α, and showed a weak positive association with 8-OHdG. On the other hand, biochemical variables showed minimal interrelations, with leukocyte and platelet counts presenting only a weak mutual correlation. Finally, global DNA methylation displayed weak positive correlations with IL-8, IL-10, TNF-α, and 8-isoprostane.

### 2.4. Bivariate Correlations Among Biomarkers, Segmented by Sex

Table 5 summarizes the significant bivariate correlations in the sex-segmented subsamples. When stratifying by sex, most of the correlation patterns observed in the total sample were retained; however, subtle yet relevant differences emerged between women and men. Among women, the inflammatory cluster remained strongly interconnected, with IL-8, IL-10, and TNF-α showing strong positive correlations (r = 0.687–0.875). IL-6 demonstrated a weak positive correlation with IL-8 and a weak negative correlation with 8-isoprostane, while IL-1β showed no significant correlations within the inflammatory profile, but it showed a weak, significant correlation with 8-OHdG. The oxidative stress biomarker 8-isoprostane correlated weakly and positively with IL-8, IL-10, TNF-α, and 8-OHdG. No associations were found between biochemical variables (leukocytes and platelets) and the remaining biomarkers in women, and global DNA methylation did not correlate with any biomarker.

In men, the inflammatory cluster also remained strongly defined, with IL-8, IL-10, and TNF-α displaying very strong positive correlations (r = 0.850–0.928). IL-1β showed a weak positive association with IL-6 and a weak negative association with 8-isoprostane, and no additional associations were detected between oxidative stress markers and the inflammatory profile. Unlike women, men showed significant associations between global DNA methylation and inflammatory biomarkers, with weak positive correlations identified for IL-8, IL-10, and TNF-α. As in women, leukocytes and platelets did not correlate with any other biomarker domain.

## 3. Discussion

In the sex-based comparison of the studied variables, illness count was higher in women than in men, reaching borderline statistical significance. This finding is consistent with previous reports indicating a higher global morbidity burden in women compared with men [2,12]. As sex-related differences have been described for specific conditions [12], our results similarly revealed differences in both self-reported and detected conditions between sexes. Women showed a higher frequency of migraine, colitis, gastritis, venous disease, thyroid disorders, anxiety, and depression, whereas men exhibited a higher frequency of detected dyslipidemia (Supplementary File). These observations are in agreement with earlier studies reporting a higher prevalence of dyslipidemia in men [13], as well as a higher prevalence of headache, anxiety, and depressive disorders in women than in men [12]. The rest of the variables that differed significantly between sexes were already discussed in the previous report derived from the same project, which focused on the association between the studied variables with global DNA methylation, unlike this study, which focused on the association between these variables with illness count [10].

In the bivariate correlations between the studied variables and the illness count, several variables showed significant associations in the total sample as well as in sex-stratified analyses; however, only a subset of these associations remained significant in the multivariate models. Among these, the sociodemographic variables age, having children, out-of-home food consumption, and excessive food consumption were positively associated with illness count in either the total sample or sex-specific multivariate analyses. In this sense, the positive association between age and illness count is consistent with previous evidence indicating that increasing age is associated with multimorbidity [14], which is expected given that aging is accompanied by progressive organ dysfunction and disease development. In contrast, the variable *having children* has been scarcely explored in relation to multimorbidity. Nevertheless, its positive association with illness count in both bivariate and multivariate analyses may reflect a higher psychosocial stress burden related to caregiving and/or greater exposure to hypercaloric dietary patterns. These factors contribute to the development of metabolic alterations, particularly dyslipidemia, which was among the most frequently reported or detected conditions in women and the most frequent in men in the present study. Along similar lines, the positive association of the variables out-of-home food consumption and excessive food consumption with illness count can be explained by the increased risk of dyslipidemia, as previously reported in a sample of Mexican university students [15]. Additionally, these dietary behaviors may also be associated with an increased risk of gastrointestinal infections, further contributing to the overall illness burden.

Other personal variables were associated with disease burden in women, either in the bivariate and/or multivariate analyses, including daily free time (negatively correlated) and monthly income (positively correlated). Regarding daily free time, previous evidence indicates that leisure-time physical activity is associated with a greater number of disease-free years [16]. Although a sedentary leisure behavior (>6.5 h/d) has been linked to an increased risk of cardiovascular disease [17], it is plausible that, in the present sample, leisure time was at least partially devoted to physically active behaviors, which may explain the observed inverse association with illness count. In contrast, the positive association between monthly income and disease burden contrasts with previous reports indicating an inverse relationship between socioeconomic position and disease burden [18]. However, comparable studies incorporating extensive adjustment for multiple confounding variables are scarce, which may partially account for this discrepancy.

In the case of psychological variables, depression and TE were positively correlated with illness count in the bivariate analyses of the women’s sample; however, among these variables, only depression remained significantly associated with illness count in the multivariate analysis in women. These findings are consistent with a previous report from our research team, in which anxiety, depression, and stress were associated with illness count in the general Mexican population [1]. They are also in line with studies showing that baseline stress reactivity of the sympatho-adrenal medullary system and the hypothalamic–pituitary–adrenal axis were related to subsequent health and disease outcomes during follow-up [19]. Additionally, the weak positive correlation between TE and illness count in the women’s multivariate analysis is supported by evidence indicating that adverse childhood experiences have been associated with the risk to develop chronic conditions, including diabetes, cardiovascular disease, respiratory disease, anxiety, and depression [20,21]. Conversely, the positive psychological variable self-motivation was negatively associated with illness count in the multivariate analyses of the global sample (with borderline statistical significance). This finding suggests that a stronger sense of purpose and motivation in life may act as a protective factor against overall disease burden.

We also observed that sleep quality was negatively associated with illness count in the multivariate analysis of the men’s sample. This finding is consistent with previous evidence describing a bidirectional relationship between multimorbidity and sleep quality [22], suggesting that high sleep quality may act as a protective factor against developing chronic conditions, while at the same time being adversely affected by existing disease burden. In addition, the positive association between systolic blood pressure (SBP) and illness count in the total and men’s multivariate analyses is consistent with the global disease burden attributable to elevated SBP, which has been reported to be higher in men than in women [23]. This association may also be partially explained by the well-established relationship between dyslipidemia and hypertension [24], both of which contribute to cardiometabolic multimorbidity.

With respect to molecular biomarkers, bivariate analyses showed that TNF-α was weakly and negatively correlated with illness count in the women’s sample, whereas 8-OHdG showed a weak positive correlation with illness count in the men’s sample. However, in the multivariate analyses, only 8-OHdG remained significantly associated with illness count in men, together with IL-6 and plateletcrit. The positive association between 8-OHdG and illness count is consistent with previous reports linking this biomarker to multiple health conditions, including cardiovascular and other inflammatory diseases [25,26], and with its well-established role as a marker of oxidative DNA damage [25]. Additionally, the positive association observed between IL-6 and illness count aligns with evidence from a recent meta-analysis reporting higher circulating IL-6 levels in elderly patients with multimorbidity compared with control groups [27], reinforcing the critical role of IL-6 in chronic inflammation [28]. Furthermore, higher values of platelet count have been associated with a higher risk of all-cause mortality and cardiovascular disease in a large Danish cohort, with a higher risk observed in men and in individuals younger than 65 years [9]. In this context, the present findings suggest that higher levels of platelet count may represent a risk factor for multimorbidity, particularly in men. Overall, these findings suggest that inflammatory and oxidative stress biomarkers, along with blood-cell-count parameters, are biomarkers of multimorbidity in men, even after adjustment for multiple covariates.

In the multivariate analyses of the total sample and the women’s subgroup, IL-10 was the only biomarker strongly and negatively associated with illness count in women. These findings coincide with previous reports describing IL-10 as a potent anti-inflammatory cytokine capable of limiting immune activation and pro-inflammatory cytokine production [29]. Moreover, IL-10 deficiency has been linked to severe, life-threatening inflammatory bowel disease in humans [29,30]. However, IL-10 has also been described as a biomarker of disease burden in multiple sclerosis [31] and as a predictor of severity and mortality in patients with acute or post-acute SARS-CoV2 infection [32]. In this context, IL-10 has been proposed to act as an endogenous danger or counter-regulatory signal released by damaged tissues in an attempt to restrain excessive inflammation [32]. In this sense, its negative correlation with illness count overall, as well as with women’s analyses, suggests that IL-10 production has a protective effect against multimorbidity, mainly in women. Finally, the lack of significant association between global DNA methylation and illness count suggests that this epigenetic marker may be more closely associated with specific diseases rather than with overall disease burden.

In the correlation analyses among the studied biomarkers, IL-10, TNF-α, and IL-8 exhibited a tight intercorrelation with highly significant correlations among them in both sexes. These findings are consistent with previous evidence demonstrating the concurrent expression of IL-8 and IL-10 in macrophages [33]. Although this pattern appears to contrast with reports describing IL-10–mediated inhibition of TNF-α gene expression [34], it has also been shown that TNF-α induces IL-10 expression [35], suggesting an inter-regulation between both cytokines. This inter-regulatory crosstalk may partly explain the high correlation among them, and is further supported by studies identifying IL-10 as a biomarker of disease burden and mortality in certain clinical conditions [31,32].

On the other hand, the low positive correlations observed among IL-6, IL-1β, and IL-8 are consistent with findings from a study conducted in middle-aged women, which reported low to moderate positive correlations between IL-6 with IL-8 and IL-1β [36]. These associations are biologically plausible given the pro-inflammatory roles of IL-6, IL-8, and 1β. Nevertheless, no other similar studies were found, and differences in the correlations between sexes may exist as those observed in this study.

Regarding oxidative stress biomarkers and their significant associations with other biological markers, we found low positive correlations between 8-isoprostane with IL-8, IL-10, and TNF-α, as well as a low negative correlation with IL-6 in women. In men, a low positive correlation was identified between 8-isoprostane and IL-1β. These findings are consistent with previous evidence indicating that 8-isoprostane can induce IL-8 expression [37]. However, a study conducted in male welders did not report significant correlations between 8-isoprostane and pro-inflammatory cytokines, including IL-8, IL-6, IL-10, and TNF-α [38]. Nevertheless, that study was limited to men and did report an association between 8-isoprostane and 8-OHdG, which is consistent with our findings in the global and women’s samples. Overall, these findings suggest the existence of a biological link between inflammatory and oxidative stress pathways as previously mentioned [39], and further suggest the presence of sex-specific differences in these associations. Finally, the results concerning the correlations between the global DNA methylation and the inflammatory and oxidative stress biomarkers were already discussed in a previous report [10].

The main limitation is the lack of reliance on self-reported data for most of the investigated diseases, which may reduce diagnostic accuracy due to potential under- or overestimation. In addition, the relatively small sample size and the large number of variables analyzed may increase random error and the risk of bias related to multiple comparisons. Another important limitation is the inclusion of acute and chronic conditions in the illness count, which could have modified the correlations obtained. Furthermore, the cross-sectional design precludes the establishment of causal relationships. Nevertheless, the comprehensive assessment of a wide range of variables enabled the identification of multiple associations that have not been previously reported. These findings provide a valuable basis for future research using larger samples and longitudinal designs to further explore and validate the observed relationships.

In conclusion, illness count was higher in women than in men and was significantly associated with a wide range of sociodemographic, biochemical, psychological, and molecular variables. Among the molecular biomarkers, IL-10, 8-OHdG, IL-6, and the plateletcrit emerged as particularly relevant, showing significant associations with illness count in the total sample and/or in sex-specific analyses. Additionally, several biomarkers exhibited significant positive correlations among them, mainly IL-10, IL-8, and TNF-α, which showed high positive correlations in both sexes, supporting the presence of coordinated inflammatory signaling. Future longitudinal and experimental studies with larger sample sizes and sex-stratified analyses are warranted to further elucidate the mechanistic relevance and clinical implications of these findings.

## 4. Materials and Methods

### 4.1. Ethical Considerations

The study was conducted in full accordance with the principles of the Declaration of Helsinki and received approval from the Research and Ethics Committee of the Health Sciences University Center (approval number CI-06123; approved on 25 September 2024). Written informed consent was obtained from all participants prior to their enrollment.

### 4.2. Subjects

Participants were eligible if they were between 18 and 60 years of age, not pregnant, and had no genetic relationship with any other individual enrolled in the study (e.g., siblings or cousins), and who lived in the urban area of Guadalajara city. Individuals were excluded if data for any study variable were missing.

#### Study Design

This research employed an observational, cross-sectional design.

### 4.3. Procedures

Recruitment and measurements were performed over a two-month period through social media announcements (e.g., WhatsApp, version 2024) and printed flyers. Additionally, students and personnel from the University Center of the Health Sciences were invited in person. Eligibility was confirmed by the research team. Individuals who agreed to participate were scheduled in a computer room at the University of Guadalajara, where they provided written informed consent and completed an electronic questionnaire (Google Forms) collecting personal information. Prior to completing the questionnaire, anthropometric assessments—including Body Mass Index (BMI) and Waist-to-Hip Ratio (WHR)—were performed, and systolic and diastolic blood pressure (SBP/DBP) were measured on the left arm using an Omron upper-arm device (HEM-7320, OMRON Healthcare CO., Ltd., Kyoto, Japan).

### 4.4. Personal and Psychological Variables

#### Personal Variables

The personal and sociodemographic variables evaluated comprised sex, age, educational attainment, employment status, parental status, presence of a romantic partner, socioeconomic level, daily time dedicated to physical activity, hours of daily leisure time, and the frequency of alcohol, tobacco, and illicit drug use. Substance use was reported on a five-point scale ranging from “never” to “four or more times per week.”

Participants also self-reported the occurrence of 28 medical conditions within the previous six months, including: diabetes mellitus (type 1 or type 2), thyroid disorders, allergies (e.g., asthma and conjunctivitis), dyslipidemia, gastritis, colitis or irritable bowel syndrome (IBS), migraine or tension-type headache, dermatologic conditions (e.g., acne and neurodermatitis), gastrointestinal infections, peptic ulcer disease, sinusitis, kidney disorders (renal failure and nephrolithiasis), anorexia/bulimia, depression or anxiety requiring medication, myocardial infarction or angina, rheumatic diseases (rheumatoid arthritis, lupus, and ankylosing spondylitis), heart failure, stroke or cerebral infarction, chronic infections (HIV, tuberculosis, long COVID, etc.), cancer (breast, cervical, prostate, and skin), leukemia or lymphoma, metastatic malignancy, venous disease (venous insufficiency and varicose veins), liver disease (hepatitis, cirrhosis, and fatty liver), chronic pulmonary disease or respiratory infections (including COVID-19), hemiplegia, and any other chronic condition requiring ongoing treatment.

Finally, the number of medications taken daily was recorded as the variable “daily drug intake.”

The presence of additional conditions was identified and incorporated into the total number of self-reported diseases (illness count) when they had not been reported by the participant: (a) dyslipidemia, defined as total cholesterol > 200 mg/dL and/or triglycerides > 150 mg/dL [40]; (b) diabetes, defined as fasting glucose > 126 mg/dL [41]; and (c) hypertension, defined as systolic blood pressure > 140 mmHg [42].

### 4.5. Psychological Variables

Psychological and emotional variables were assessed using a battery of standardized instruments. Perceived stress was evaluated with the 10-item Perceived Stress Scale (PSS-10) [43,44], while symptoms of depression and anxiety were measured using the Patient Health Questionnaire (PHQ-9) [45] and the Generalized Anxiety Disorder Scale (GAD-7) [46], respectively. Psychological well-being was assessed through Ryff’s Psychological Well-Being Scale (PWBS), which captures six core dimensions: self-acceptance, positive relations with others, autonomy, environmental mastery, purpose in life, and personal growth [47,48]. Sense of coherence was measured with the 13-item Sense of Coherence Scale (SOC-13), which comprises the dimensions of comprehensibility, manageability, and meaningfulness [49]. Emotional intelligence—specifically assertiveness, emotion identification, and self-motivation—was evaluated using the corresponding subscales of the Trait Emotional Intelligence Questionnaire (TEIQue), each consisting of 5 to 6 items (Supplementary File) [50]. Positive emotions were assessed with the Positivity Self-Test [51]. Finally, exposure to traumatic events (TE) was measured with the Cumulative Lifetime Adversity Scale, which records the occurrence of 37 traumatic events using four frequency categories ranging from “never” to “more than twice” [52].

### 4.6. Lifestyle Scales

Sleep quality was assessed using the second item of the OVIEDO Sleep Questionnaire, which includes five items rated on a scale ranging from 0 (poor quality) to 4 (excellent quality). Sleep satisfaction was measured using the first item of the OVIEDO Sleep Questionnaire, with response categories ranging from 1 (very dissatisfied) to 7 (very satisfied) [53]. Additionally, dietary quality was evaluated using the Mini-ECCA survey, a brief instrument validated for use in the Mexican population [54]. From the Eating Behavioral Questionnaire [55], we included two ordinal questions: (a) out-of-home food consumption and (b) frequency of excessive food consumption; both items had 7 options, ranging from 1 (less than once in a month) to 7 (all the days of the month).

### 4.7. Collection of Venous Blood Samples

Fasting blood samples were drawn by venipuncture in the early morning from all participants, who had abstained from food for a minimum of 8 h. Once collected, the samples were immediately processed for biochemical analyses.

### 4.8. Assessment of Biochemical Parameters

Biochemical analyses were conducted on venous blood samples from all participants. Total cholesterol, glucose, and urea were quantified using colorimetric methods on the H-100 Automated Clinical Chemistry Analyzer (Model H100, HLAB Supply Ltd., Denver, CO, USA). Complete blood count parameters—hemoglobin, hematocrit, platelet count, plateletcrit, total leukocytes, and leukocyte subpopulations—were assessed using an electronic impedance variation system (HORIBA ABX Micros ES 60 Hematology Analyzer, Horiba Ltd. Montpellier, Grabels, France).

### 4.9. Analysis of Serum Inflammatory and Oxidative Stress Biomarkers

Serum biomarker concentrations were quantified using enzyme-linked immunosorbent assays (ELISA), conducted according to the manufacturer’s protocols for each kit. All serum samples were stored at −80 °C until analysis. The following analytes were measured using MyBioSource kits (MyBioSource Inc., San Diego, CA, USA): IL-1β (MBS263843), IL-6 (MBS021993), IL-8 (MBS763092), IL-10 (MBS764410), 8-isoprostane (MBS3802509), 8-hydroxy-2′-deoxyguanosine (MBS267161), and TNF-α (catalog MBS175820).

### 4.10. DNA Extraction

Genomic DNA was isolated from peripheral blood leukocytes using a modified Miller and CTAB/DTAB protocol. DNA concentration (A260) and purity (A260/A280 ratio) were assessed with a NanoDrop spectrophotometer (NanoDrop Technologies Inc., Delaware, TX, USA). The extracted DNA was subsequently diluted in Tris-EDTA buffer to a final concentration of 100 ng/µL.

### 4.11. Determination of Global DNA Methylation Levels

Global DNA methylation was assessed in duplicate using 100 ng of leukocyte-derived DNA. Levels of 5-methylcytosine (5-mC) were measured through a colorimetric ELISA, employing specific capture and detection antibodies and quantifying absorbance at 450 nm on a Multiskan™ FC microplate spectrophotometer (Thermo Fisher Scientific, Waltham, MA, USA). Optical density values were proportional to the degree of methylation. Quantification procedures adhered to the manufacturer’s instructions for the MethylFlash™ Methylated DNA Quantification Kit (Epigentek, P-1030; Farmingdale, NY, USA).

### 4.12. Statistical Analysis

Continuous variables were summarized as mean and standard deviation when their distribution approximated normality, and as median with ranges when they were non-normally distributed; categorical variables were described using frequencies and percentages. Sex-related differences in sociodemographic characteristics were examined using chi-square tests for categorical variables and Student’s *t* test or the Mann–Whitney *U* test for continuous variables, depending on distributional assumptions.

Associations of illness count with psychological, biochemical, anthropometric, global DNA methylation, and physiological measures—as well as inflammatory and oxidative stress biomarkers—were assessed using Pearson or Spearman correlation coefficients, according to the normality of each variable.

To identify factors independently associated with illness count, we conducted multiple linear regression analyses using a stepwise selection procedure. In this model, the dependent variable was continuous (illness count: non-normal distribution), and the pool of candidate predictors included continuous, dichotomous, and ordinal variables. Analyses were performed in the overall sample and stratified by sex to account for potential confounding, and *p*-values reported from multivariable models represent adjusted estimates; for each model, we obtained the R and R^2^ values, as well as the adjusted R values for each model. This last value was obtained to adjust for overfitting. The biomarker outliers were calculated by obtaining the number of values that fall outside the following range: The upper limit was obtained by adding 1.5 times the interquartile range to the third quartile, and the lower limit was obtained by subtracting this value from the first quartile.

All statistical analyses were carried out using SPSS (version 31), JASP (version 0.95.1), and a two-tailed *p*-value < 0.05 was considered statistically significant.

## Figures and Tables

**Table 1:** Comparison of studied variables between sexes.

Variables	Female n = 83	Male n = 74	*p*
**Sociodemographic**			
Age (years), median (range)	23.0 (18–58)	25.5 (18–54)	0.535
Highest education level, n (%)			**0.019**
- Middle school	4 (4.8)	3 (4.1)	
- High school	41 (49.4)	25 (33.8)	
- Bachelor’s degree	35 (42.2)	31 (41.8)	
- Master’s degree	2 (2.4)	9 (12.2)	
Doctorate (Ph.D.)	1 (1.2)	6 (8.1)	
With a romantic partner, n (%)	42 (50.6)	46 (62.2)	0.145
Having children, n (%)	22 (26.5)	22 (29.7)	0.653
Employed, n (%)	52 (62.7)	53 (71.6)	0.233
Monthly income (MXN), n (%)			**<0.001**
- Low	7 (8.4)	1 (1.4)	
- Lower-middle	33 (39.8)	15 (20.3)	
- Upper-middle	33 (39.8)	28 (37.7)	
- High	6 (7.2)	19 (25.7)	
- Very high	4 (4.8)	11 (14.9)	
Free time (hours), median (range)	4.0 (0–12.0)	4.0 (0–12.0)	0.077
Daily exercise (hours), n (%)			0.476
- 0 to 0.5	35 (42.2)	29 (39.2)	
- 1.0 to 1.5	34 (41.0)	25 (33.8)	
- 2.0 to 2.5	10 (12.0)	14 (18.9)	
- >3	4 (4.8)	6 (8.1)	
Frequency of out-of-home food consumption, n (%)			**0.030**
- Less than once a month	5 (6.0)	1 (1.3)	
- Once a month	3 (3.6)	3 (4.0)	
- Once every 15 days	11 (13.3)	14 (18.9)	
- Once to twice a week	39 (47.0)	19 (25.7)	
- Three to four times a week	15 (18.1)	15 (20.3)	
- Five to six times a week	3 (3.6)	7 (9.5)	
- Every day	7 (8.4)	15 (20.3)	
Frequency of excessive food consumption, n (%)			0.574
- Less than once a month	12 (14.5)	6 (8.1)	
- Once a month	8 (9.6)	11 (14.9)	
- Once every 15 days	19 (22.9)	12 (16.1)	
- Once to twice a week	25 (30.1)	21 (28.4)	
- Three to four times a week	12 (14.5)	17 (23.0)	
- Five to six times a week	3 (3.6)	3 (4.0)	
- Every day	4 (4.8)	4 (5.4)	
Weekly dietary supplement use, n (%)			0.161
- Never	41 (49.4)	46 (62.1)	
- Less than once	16 (19.3)	15 (20.3)	
- 1 to 2	8 (9.6)	4 (5.4)	
- 3 to 4	6 (7.2)	6 (8.1)	
- Daily	12 (14.5)	3 (4.1)	
Total number of illnesses, median (range)	4.0 (0–12)	4 (0–9)	**0.075**
Daily intake of drugs	0.0 (0.0–4.0)	0.0 (0.0–3.0)	0.081
Sleep quality, median (range)	2.8 (0.4–4.0)	3.2 (0.0–4.0)	0.122
Alcohol use frequency, n (%)			**0.009**
- Never	12 (14.5)	11 (14.9)	
- 2 to 4 times per year	27 (32.5)	14 (18.9)	
- Once a month or less	36 (43.4)	27 (36.4)	
- 2 to 3 times per week	6 (7.2)	21 (28.4)	
- 4 or more times per week	2 (2.4)	1 (1.4)	
Smoking frequency, n (%)			0.369
- Never	71 (85.6)	59 (79.6)	
- 2 to 4 per year	5 (6.0)	3 (4.1)	
- At least one monthly	2 (2.4)	3 (4.1)	
- 2 to 3 weekly	3 (3.6)	2 (2.7)	
- >4 weekly	2 (2.4)	7 (9.5)	
Consumption of the seven evaluated illicit substances, Mean ± SD	0.0 (0.0–0.3)	0.0 (0.0–0.7)	0.091
**Psychological**			
Positive emotions, mean ± SD	2.6 ± 0.8	2.9 ± 0.6	**0.015**
TEIQUE: Assertiveness, mean ± SD	4.5 ± 1.2	4.8 ± 1.3	0.183
TEIQUE: Emotion identification, median (range)	5.4 (1.2–7.0)	5.4 (1.2–7.0)	0.931
TEIQUE: Self-motivation, median (range)	5.2 (1.6–7.0)	5.2 (2.2–7.0)	0.272
SOC_13: Comprehensibility, median (range)	4.2 (1.6–6.6)	4.8 (2.0–7.0)	**0.033**
SOC_13: Manageability, mean ± SD	4.1 ± 1.4	4.5 ± 1.4	0.078
SOC_13: Meaningfulness, median (range)	5.3 (1.8–7.0)	5.3 (2.8–7.0)	0.895
GAD-7, median (range)	1.0 (0.0–3.0)	0.9 (0.0–3.0)	0.405
Dietary quality (Mini-ECCA), median (range)	8.0 (3.0–12.0)	6.5 (2.0–12.0)	0.083
PHQ-9, median (range)	0.7 (0.0–2.7)	0.4 (0.0–2.1)	**0.029**
PSS-10, mean ± SD	1.6 ± 0.8	1.4 ± 0.6	**0.048**
Traumatic events in life, median (range)	0.4 (0.0–1.2)	0.4 (0.0–2.0)	0.605
PWBS_Self-acceptance, median (range)	4.8 (1.0–6.0)	5.0 (2.0–6.0)	0.075
PWBS_Autonomy, median (range)	4.2 (1.5–6.0)	4.5 (2.2–6.0)	**0.020**
PWBS_Environmental mastery, Mean ± SD	4.47 ± 1.1	4.71 ± 0.9	0.136
PWBS_Positive relations, median (range)	5.0 (1.0–6.0)	4.7 (1.8–6.0)	0.475
PWBS_Life purpose, median (range)	4.8 (1.4–6.0)	5.0 (1.6–6.0)	0.101
PWBS_Personal growth, median (range)	5.7 (1.3–6.0)	5.7 (3.0–6.0)	0.323
**Biochemicals**			
Erythrocytes (1 × 10^3^/µL), median (range)	4.2 (3.0–5.9)	4.8 (4.1–6.6)	**<0.001**
Leukocytes (1 × 10^3^/µL), median (range)	5.9 (3.4–11.0)	5.8 (3.5–8.5)	0.524
Lymphocytes (1 × 10^3^/µL), median (range)	1.7 (0.9–3.0)	1.8 (0.8–3.5)	0.456
Monocytes (1 × 10^3^/µL), median (range)	0.2 (0.1–0.4)	0.2 (0.1–0.4)	0.105
Granulocytes (1 × 10^3^/µL), median (range)	3.9 (2.0–7.9)	3.8 (1.9–6.3)	0.187
Platelets (1 × 10^3^/µL), mean ± SD	236.5 ± 48.9	209.4 ± 40.7	**<0.001**
Plateletcrit, mean ± SD	0.20 ± 0.04	0.17 ± 0.03	**<0.001**
Hemoglobin (g/dL), median (range)	12.7 (7.3–17.4)	14.5 (12.9–19.2)	**<0.001**
Hematocrit, mean ± SD	43.1 ± 4.23	50.5 ± 3.27	**<0.001**
Cholesterol (mg/dL), mean ± SD	180.8 ± 33.7	189.9 ± 42.1	0.140
Glucose (mg/dL), median (range)	83.8 (59.4–120.6)	92.3 (61.6–232.0)	**<0.001**
Urea (mg/dL), median (range)	26.2 (14.2–143.7)	27.0 (10.0–58.7)	0.306
Global DNA methylation (%), median (range)	0.4 (0.0–1.9)	0.5 (0.1–2.1)	**0.045**
**Levels of inflammatory and oxidative stress biomarkers**			
TNF-α (pg/mL), median (range)	62.9 (15.6–1000.0)	403.5 (15.6–1000.0)	0.132
IL-8 (pg/mL), median (range)	9.8 (7.8–462.0)	9.4 (7.8–380.5)	0.259
IL-6 (pg/mL), median (range)	6.2 (3.1–329.0)	4.2 (3.1–23.3)	**0.006**
IL-1β (pg/mL), median (range)	13.7 (7.8–1000.0)	13.3 (8.3–27.4)	0.410
IL-10 (pg/mL), median (range)	43.3 (7.8–1741.6)	105.8 (7.8–1741.6)	0.399
8-Isoprostane (pg/mL), median (range)	291.0 (230.1–418.3)	300.9 (246.4–364.4)	0.175
8-OHdG, median (range)	1.9 (0.6–10.0)	2.0 (1.1–2.9)	0.218
**Anthropometrics and blood pressure**			
BMI, median (range)	25.9 (16.4–39.9)	25.2 (18.7–38.9)	0.783
WHR, median (range)	0.8 (0.7–1.0)	0.9 (0.7–1.2)	**<0.001**
Systolic BP (mmHg), median (range)	106.0 (80.0–155.0)	120.0 (90.0–164.0)	**<0.001**
Diastolic BP (mmHg), median (range)	75.0 (57.0–106.0)	79.0 (62.0–120.0)	**0.025**

Quantitative variables are presented as median with range or as mean ± standard deviation (SD), whereas qualitative variables are reported as frequencies and percentages. Sex comparisons were performed using Chi-square or Fisher’s exact test for categorical variables, and Student’s *t* test or Mann–Whitney *U* test for continuous variables, as appropriate. Statistically significant differences were defined as *p* < 0.05 and were highlighted in bold. Monthly income was categorized into five levels (from low to very high). Smoking and alcohol consumption frequency ranged from 0 to 4 (never to more than four times per week). Sleep quality (OVIEDO scale) ranged from 0 to 4 (poor to excellent). Positive emotions (PSS scale) ranged from 1 to 5 (never to almost always). Emotional intelligence subscales (TEIQue)—assertiveness, emotion identification, and self-motivation—were rated from 1 to 7 (strongly disagree to strongly agree). Sense of coherence (SOC-13) subscales—comprehensibility, manageability, and meaningfulness—were scored from 1 to 7 (never or almost never to frequently). Anxiety symptoms (GAD-7) ranged from 0 to 3 (never to nearly every day). Dietary quality (Mini-ECCA) ranged from 0 to 12 (very low to very high). Depressive symptoms (PHQ-9) were scored from 0 to 3 (very low to very high). Perceived stress (PSS-10) ranged from 0 to 3 (never to frequently). Traumatic life events represent an average frequency of 37 events, scored from 0 to 3 (never to more than twice). Psychological well-being subscales (PWBSs)—self-acceptance, autonomy, environmental mastery, positive relations, life purpose, and personal growth—were scored from 1 to 6 (strongly disagree to strongly agree). TNF-α = tumor necrosis factor alpha; IL = interleukin; 8-OHdG = 8-hydroxy-2-deoxyguanosine; BMI = body mass index; WHR = waist-to-hip ratio; and BP = blood pressure.

**Table 2:** Significant bivariate correlations between the studied variables and illness count.

Variables	Total Sample n = 157	Women n = 83	Men n = 74
**Sociodemographic**			
Sex	−0.142 ^α^	—	—
Having children	0.222 **	—	0.336 **
Age	0.180 *	—	0.410 **
Schooling	—	—	0.218 ^α^
Free time	−0.190 *	−0.209 ^α^	—
Out-of-home food consumption	0.174 *	0.240 *	—
Excessive food consumption	0.208 **	0.242 *	0.200 ^α^
Dietary quality	—	—	−0.206 ^α^
BMI	0.248 **	—	0.399 **
WHR	—	—	0.381 **
**Biochemicals**			
Erythrocytes	—	—	0.217 ^α^
Hematocrit	—	—	0.219 ^α^
**Psychological**			
Depression (PHQ-9)	—	0.286 *	—
Traumatic events (TE)	—	0.245 *	—
**Biomarkers variables**			
TNF-α	—	−0.218 *	—
8-OHdG	—	—	0.251 *

* *p* < 0.05, ** *p* < 0.01. Correlations were calculated with Spearman correlation tests. GAD-7: generalized anxiety disorder; PHQ-9: depression with patient-health questionnaire; TE: traumatic events in life; BMI: body mass index, WHR: Waist-to-hip ratio. ^α^ Borderline *p*-value. TNF-α = tumor necrosis factor alpha, 8-OHdG = 8-hydroxy-2-deoxyguanosine.

**Table 3:** Multivariate regression analysis for illness counts in overall sample and stratified by sex.

Variables	B	Beta Coefficient	Significance	Change in R^2^	Tolerance
**Total sample**					
Constant	3.384	—	0.051	—	—
With children	0.956	0.166	0.040	0.062	0.900
Male sex	−1.591	−0.316	<0.001	0.044	0.719
Out-of-home food consumption	0.329	0.191	0.017	0.035	0.922
IL-10	−0.001	−0.210	0.008	0.033	0.937
Systolic blood pressure (SPB)	0.031	0.171	0.056	0.020	0.731
Self-motivation	−0.275	−0.142	0.066	0.020	0.980
**Women**					
Constant	4.306	—	<0.001	—	—
IL-10	−0.001	−0.290	0.005	0.091	0.996
Free time	−0.421	−0.364	<0.001	0.072	0.891
Monthly income	0.952	−0.333	0.004	0.051	0.809
Depression (PHQ-9)	1.258	0.290	0.007	0.076	0.906
**Men**					
Constant	−15.269	—	<0.001	—	—
Age	0.094	0.416	<0.001	0.135	0.816
8-OHdG	1.398	0.262	0.009	0.064	0.965
Systolic blood pressure (SBP)	0.068	0.359	<0.001	0.069	0.922
IL-6	0.108	0.208	0.040	0.061	0.937
Plateletcrit	20.265	0.256	0.013	0.041	0.921
PWBS: Positive relations	0.489	0.250	0.015	0.048	0.923
Sleep quality	−0.455	−0.195	0.064	0.033	0.854

The samples for each multivariate analysis are highlighted in bold. R and adjusted R values of the models: total: 0.462, 0.423; women: 0.538, 0.500; and men: 0.671, 0.620.

**Table 4:** Significant bivariate correlations between biomarkers in total sample.

Variables	IL-1β	IL-6	IL-8	IL-10	TNF-α	8-Isoprostane	8-OHdG	Leukocytes	Platelets	% Global DNA Methylation
**Inflammatory biomarkers**										
IL-1β	—	0.237 **	—	—	—	—	0.257 **	—	—	—
IL-6	0.237 **	—	0.168 *	—	—	−0.268 **	—	—	—	—
IL-8	—	0.168 *		0.793 *	0.751 **	0.184 *	—	—	—	0.265 **
IL-10	—	—	0.793 *	—	0.902 **	0.184 *	—	—	—	0.211 **
TNF-α	—	—	0.751 **	0.902 **	—	0.254 **	—	—	—	0.210 **
**Stress oxidative biomarkers**										
8-Isoprostane	—	−0.268 **	0.184 *	0.184 *	0.254 **	—	0.265 **	—	—	0.160 *
8-OHdG	0.257 **	—	—	—	—	0.265 **	—	—	—	—
**Biochemical variables**										
Leukocytes	—	—	—	—	—	—	—	—	0.180 *	—
Platelets	—	—	—	—	—	—	—	0.180 *	—	—
% Global DNA methylation	—	—	0.265 **	0.211 *	0.210 **	0.160 *	—	—	—	—

The type of variables are highlighted in bold. *, *p* < 0.05, and **, *p* < 0.01. Correlations were performed with the Spearman correlation test. IL: interleukin; TNF-α: tumor necrosis factor alpha; 8-hydroxy-2-deoxyguanosine.

**Table 5:** Significant bivariate correlations between biomarkers and sexes.

Variables	Sex	IL-1β	IL-6	IL-8	IL-10	TNF-α	8-Isoprostane	8-OHdG	Leukocytes	Platelets	% Global DNA Methylation
**Inflammatory biomarkers**											
IL-1β	F	—	—	—	—	—	—	0.358 **	—	—	—
	M	—	0.268 *	—	—	—	0.258 *	—	—	—	—
IL-6	F	—	—	0.246 *	—	—	−0.306 **	—	—	—	—
	M	0.268 *	—	—	—	—	—	—	—	—	—
IL-8	F	—	0.246 *	—	0.750 **	0.687 **	0.274 *	—	—	—	—
	M	—	—	—	0.856 **	0.850 **	—	—	—	—	0.368 *
IL-10	F	—	—	0.750 **	—	0.875 **	0.268 *	—	—	—	—
	M	—	—	0.856 **	—	0.928 **	—	—	—	—	0.268 *
TNF-α	F	—	—	0.687 **	0.875 **	—	0.349 **	—	—	—	—
	M	—	—	0.850 **	0.928 **	—	—	—	—	—	0.252 *
**Stress oxidative biomarkers**											
8-Isoprostane	F	—	−0.306 **	0.274 *	0.268 *	0.349 **	—	0.307 **	—	—	—
	M	−0.258 *	—	—	—	—	—	—	—	—	—
8-OHdG	F	0.358 **	—	—	—	—	0.307 **	—	—	—	—
	M	—	—	—	—	—	—	—	—	—	—
**Biochemical variables**											
Leukocytes	F	—	—	—	—	—	—	—	—	—	—
	M	—	—	—	—	—	—	—	—	—	—
Platelets	F	—	—	—	—	—	—	—	—	—	—
	M	—	—	—	—	—	—	—	—	—	—
% Global DNA methylation	F	—	—	—	—	—	—	—	—	—	—
	M	—	—	0.368 **	0.268 *	0.256 *	—	—	—	—	—

The biomarkers and the types of the variables analyzed are hightlighted in bold. The background color helps to visualize each biomarker. *, *p* < 0.05, **, *p* < 0.01. Correlations were performed with the Spearman correlation test. IL: interleukin; TNF-α: tumor necrosis factor alpha; 8-hydroxy-2-deoxyguanosine.

## Data Availability

The original contributions presented in this study are included in the article/Supplementary Materials. Further inquiries can be directed to the corresponding authors.

## Supplementary Materials

The following supporting information can be downloaded at: https://www.mdpi.com/article/10.3390/ijms27052408/s1.
