# Supplementary material for: Association Between Personal, Behavioral, Psychological, Biochemical and Molecular Biomarkers with Illness Count in a Sample of Mexican Individuals
Source: Int J Mol Sci. 2026 Mar 5;27(5):2408. doi: 10.3390/ijms27052408 (PMC12985521; doi:10.3390/ijms27052408)
Supplement: Supplementary file 1 [file ijms-27-02408-s001.zip › ijms-4156565-supplementary.pdf]

Supplementary file 1

Items included in the emotional intelligence subscales of the TEIQUE scale

Instruction: Please indicate the degree of agreement or disagreement with the following statements.
Answer options: 1: absolutely disagree- 7: absolutely agree

Self-motivation

1.- On the whole, I'm a highly motivated person

2.- Sometimes, it feels like I'm producing a lot of good work effortlessly

3.- I normally find it difficult to keep myself motivated

4.- I tend to get a lot of pleasure just from doing something well

5.- I lose interest in what I do quite easily

Emotion perception

1.- I often find it difficult to recognize what emotion I am feeling

2.- I´m never really sure what I´m feeling

3.- Many times, I can´t figure out what emotion I´m feeling

4.- On the whole, I find it difficult to describe my feelings

5.- Most of the time, I know exactly why I feel the way I do

Assertiveness

1.- When I disagree with someone, I usually find it easy to say so

2.- I tend to "back down" even if I know I´m right

3.- I usually find it difficult to express myself clearly

4.- I would normally defend my opinions even if it meant arguing with important people

5.- I tend to speak well and clearly

6.- I often find it difficult to stand up for my rights

Supplementary file 2. Self-reported morbidity over the previous six months and moribidity conditions detected during the study.

Disease, n (%)	Women n=83	Detected	Men n=74	Detected
Hypertension	2 (2.4)	2 (2.4)	0 (0.0)	4 (5.4)
Diabetes mellitus (type 1 or 2)	2 (2.4)	1 (1.2)	2 (2.7)	1 (1.4)
Thyroid disease	5 (6.0)	-	0 (0.0)	-
Allergies (e.g., asthma, conjunctivitis)	19 (22.9)	-	13 (17.6)	-
Dyslipidemia	12 (14.5)	24 (28.9)	5 (6.8)	46 (62.1)
Gastritis	34 (41.0)	-	22 (29.7)	-
Colitis/irritable bowel syndrome	34 (41.0)	-	17 (23.0)	-
Migraine or tension headache	44 (53.0)	-	22 (29.7)	-
Dermatologic problems (acne, neurodermatitis)	30 (36.1)	-	21 (28.4)	-
Gastrointestinal infections	25 (30.1)	-	20 (27.0)	-
Peptic ulcer disease	2 (2.4)	-	5 (6.8)	-
Sinusitis	9 (10.8)	-	11 (14.9)	-
kidney disease (renal failure, nephrolithiasis)	3 (3.6)	-	0 (0.0)	-
Anorexia/bulimia	3 (3.6)	-	0 (0.0)	-
Depression requiring medication	6 (7.2)	-	1 (1.4)	-
Anxiety requiring medication	10 (12.0)	-	2 (2.7)	-
Myocardial infarction/angina	1 (1.2)	-	1 (1.4)	-
Rheumatic diseases (rheumatoid arthritis, lupus, ankylosing spondylitis)	0 (0.0)	-	4 (5.4)	-
Heart failure	0 (0.0)	-	0 (0.0)	-
Stroke/cerebral infarction	0 (0.0)	-	0 (0.0)	-
Chronic infections (HIV, tuberculosis, long COVID, etc.)	1 (1.2)	-	2 (2.7)	-
Cancer (breast, cervical, prostate, skin)	0 (0.0)	-	0 (0.0)	-
Leukemia/lymphoma	0 (0.0)	-	0 (0.0)	-
Advanced cancer (metastatic)	0 (0.0)	-	0 (0.0)	-
Venous disease (venous insufficiency, varicosities)	14 (16.9)	-	1 (1.4)	-

Liver disease (hepatitis, cirrhosis, fatty liver)	3 (3.6)	-	1 (1.4)	-
Chronic lung disease	0 (0.0)	-	0 (0.0)	-
Respiratory infections (including COVID)	14 (16.9)	-	14 (18.9)	-
Hemiplegia (paralysis)	0 (0.0)	-	0 (0.0)	-
Any other chronic illness requiring ongoing treatment	7 (8.4)	-	3 (4.1)	-
